# A systematic review of ecological momentary assessment in autism research

**DOI:** 10.1177/13623613241305722

**Published:** 2024-12-18

**Authors:** Yixin Chen, Zhenyang Xi, Talya Greene, Will Mandy

**Affiliations:** University College London, London, UK

**Keywords:** autism spectrum disorders, ecological momentary assessment, experience sampling method, systematic review

## Abstract

Ecological momentary assessment has been increasingly used in autism studies over the last decade to capture and understand autistic people’s behaviours, thoughts, feelings and daily experiences. This systematic review synthesised previous autism ecological momentary assessment studies to learn about the feasibility of ecological momentary assessment with autistic people and derive ideas to optimise the applicability of ecological momentary assessment in autism studies. Five databases (MEDLINE, PsycINFO, Web of Science, EMBASE and CINAHL) were searched, covering the time between January 1990 and October 2024, for studies using ecological momentary assessment with autistic participants. We identified 32 studies with 930 autistic participants. These studies supported the acceptability and feasibility of ecological momentary assessment with young and middle-aged autistic adults with average or above-average intelligence and language skills. Previous researchers and autistic participants in the reviewed literature suggested considering adaptations in the design of sampling schedules, ecological momentary assessment questionnaires and the implementation of incentive strategies, to improve the applicability of ecological momentary assessment with the autistic population. Future ecological momentary assessment studies could address the limitations in the extant literature, for example, lack of evidence from youth and older autistic people and a wider range of study topics.

**Lay abstract:**

Ecological momentary assessments assess people’s in-the-moment thoughts and behaviours in their daily lives in natural environments. The number of ecological momentary assessment studies with autistic people has increased over the last decade. For the first time, this review (1) summarises how well ecological momentary assessment works for allowing autistic people to describe and express their thoughts, emotions and experiences, and (2) provides suggestions for the design of ecological momentary assessment to make this research method more accessible to future autistic participants. In total, we synthesised participation experiences from 930 autistic people. Overall, ecological momentary assessment is generally acceptable for autistic adults aged from 18 to 60 and with average or above-average intelligence and language. We also identified several issues in the ecological momentary assessment procedure and suggested researchers consider these when designing future ecological momentary assessment studies with autistic people. The findings of this review provide evidence that ecological momentary assessment can be used to investigate many different questions with autistic people and suggest a wider application of ecological momentary assessment in future studies with autistic people.

## Introduction

Autism research commonly seeks to measure autistic people’s behaviours, thoughts, feelings and experiences. To this end, a wide range of data collection methods have been employed. One available method is ecological momentary assessment (EMA), alternatively known as the experience sampling method (ESM), which has been widely used in studies with both clinical (e.g. depressive disorder by Colombo et al. (2019); anxiety disorder by Walz et al. (2014); eating disorder by Schaefer et al. (2020)) and non-clinical (e.g. mental health and behaviour by Huckins et al. (2020); sleep and affect by Shen et al. (2022)) samples. EMA refers to a structured diary method for participants to self-report their thoughts, feelings, symptoms, context (e.g. location, company, activity) and the appraisal of the context in daily life. Participants in EMA studies are providing data in the real world and in real time (Myin-Germeys et al., 2009; Myin-Germeys & Kuppens, 2022). The reports typically have to be filled out several times a day during several consecutive days (Myin-Germeys et al., 2003). In this review, we focused on the implementation of EMA with the autistic population.

The number of autism EMA studies has increased over the last decade, reflecting the potential of this method to provide insights into the experiences and perspectives of autistic people. EMA has some specific methodological and research advantages compared with traditional retrospective or observational methods of data collection in autism research. First, compared with one-off questionnaires or observational assessments, ‘in-the-moment’ experiences or experiences over relatively short time periods (e.g. minutes or hours), collected repeatedly through EMA measures, likely have less response biases associated with retrospective recall (Coyne & Gottlieb, 1996; Trull & Ebner-Priemer, 2009). Second, the self-report nature of EMA reduces biases associated with over-reliance on information from others. Considering the possible mismatch between autistic people’s internal feelings and thoughts, and the information being captured and recorded by observers, it is necessary to understand what autistic people experience by themselves, rather than through others’ perspectives. Third, as a real-world assessment, EMA has improved ecological validity compared with assessments conducted in a laboratory setting. Autistic people’s subjective experiences within natural settings may not be recreated or detected in a laboratory environment (Myin-Germeys et al., 2009; Shiffman et al., 2008; Trull & Ebner-Priemer, 2009), such as by Autism Diagnostic Observation Schedule (ADOS-2; Lord et al., 2012). However, rather than assuming EMA data are superior (see Feller et al., 2024), it should be noted that different types of assessments can all collect valuable information, and as such, we could view these assessments as complementary in understanding people’s subjective experiences (Solhan et al., 2009). Furthermore, EMA in autism studies has potential to gain insights into the dynamics of autistic people’s behaviours/thoughts which may not be effectively captured by commonly used one-off retrospective methods and observational assessments. Although making conclusive causal interpretations is unwarranted from EMA measures, the temporal directionality of the predictive relationship between autistic people’s behaviours or emotional/affect states hints at the sequence of these subjective experiences, thereby helping contribute to the drawing of causal inferences (Myin-Germeys & Kuppens, 2022). Consequently, EMA has been increasingly used in autism studies and will likely continue to be applied to further understand outcomes that emerge over time.

With these methodological and research advantages of EMA in autism research, existing autism studies have implemented EMA to explore diverse study topics. However, although EMA has been conducted with people across the lifespan (Myin-Germeys & Kuppens, 2022; Rah et al., 2006; Schmiedek et al., 2010), with mild intellectual disability (Gosens et al., 2024; Hulsmans et al., 2023; Wilson et al., 2020) and with mental health conditions (e.g. Colombo et al., 2019), the feasibility of EMA with autistic populations has not been systematically reviewed. Given the heterogeneity of autism, whether EMA is feasible for autistic people with different demographic, clinical and intellectual characteristics remains uncertain.

Regarding the research topics in autism EMA studies, several individual empirical studies have examined the feasibility, reliability and validity of EMA and suggested that autistic people’s social and daily experience (Chen et al., 2014, 2015), stressors (Khor, Gray, et al., 2014) and leisure participation (Song et al., 2023) can be effectively captured by EMA measures. However, it should be noted that each of the conclusions about the feasibility of EMA was for a single autism research area and from one autistic sample group. In the current review, the included autism EMA studies were not limited by research area or autistic participants’ demographic, clinical and intellectual characteristics. For the first time, we systematically reviewed the implementation of EMA in the autism literature, aiming to explore the feasibility of EMA measures across autism research topics and autistic populations.

Considering participants’ characteristics and study aims, many EMA studies have adapted the designs of EMA measures. Given the lack of shared knowledge of the designs that could possibly limit the applicability of EMA with autistic participants, there is enormous heterogeneity in the methodologies and procedures in previous autism EMA studies. Autistic participants and researchers have commented on some previous EMA designs, both positively and negatively, and co-produced suggestions for future implementation of EMA with autistic people (e.g. Cooper et al., 2022; Cordier et al., 2016; Dallman et al., 2022). Several previous empirical studies and reviews that do not focus on autism research have examined how EMA designs influence participants’ responses (Eisele et al., 2022; Hasselhorn et al., 2022; Wrzus & Neubauer, 2023) and suggested some practical recommendations for future EMA studies (van Roekel et al., 2019). To further map autism research needs, in addition to synthesising the methodological characteristics of EMA across previous autism studies, we aimed to summarise EMA designs that could be considered to improve the applicability of EMA with autism. Although there is no one right answer for EMA designs (Myin-Germeys & Kuppens, 2022), this review could be a useful resource for researchers designing future autism EMA studies.

To summarise, in the current review, we synthesised previous autism EMA studies to learn about the feasibility of EMA with autistic people and derive ideas to optimise the applicability of EMA in autism studies. To achieve this aim, we had the following objectives:

1. To summarise the study aims and participant characteristics of autism EMA studies.
2. To summarise the methodological characteristics and questionnaire designs of autism EMA studies.
3. To identify the feasibility of EMA in autism research, as indexed by response rates and autistic participants’ qualitative feedback.
4. To summarise implementation challenges, adaptations of EMA methodologies, and recommendations for future autism EMA studies proposed by researchers and autistic participants.

We valued autistic participants’ views on their EMA participation experience. Thus, autistic people’s views about the methodologies and procedures that were collected before (i.e. participatory research) and after (i.e. feedback) EMA studies were considered when addressing the above four research objectives.

## Method

This systematic review was reported according to the Preferred Reporting Items for Systematic Reviews and Meta-analyses (PRISMA) standards (Page et al., 2021). The protocol was registered with the International Prospective Register of Systematic Reviews (PROSPERO), registration number CRD42023421476. Any deviations from the protocol are noted below.

### Search strategy

The literature search was conducted on English studies published on MEDLINE (Ovid), PsycINFO (Ovid), Web of Science Core Collection (Web of Science), EMBASE (Ovid) and CINAHL (EBSCOhost) databases between January 1990 and October 2024. The search terms were developed by the first author, developed from search terms used in prior relevant reviews (e.g. Bos et al., 2015). Similar search terms were used for all database searches. The search terms combined text words and MeSh terms/subject headings (depending on the databases). The search focused on two main areas: autism spectrum disorder (example terms: autis*, asperger*) and method (example terms: ecological momentary assessment, experience sampling). The text words were searched in title/abstract/keywords to reduce the number of unqualified records. The full search strategy and limits applied to the search strategy are provided in supplementary material.

### Eligibility criteria

Three inclusion criteria were applied.

1. Participant characteristics: Participants should have reported a diagnosis of autism spectrum disorder according to the *Diagnostic and Statistical Manual of Mental Disorders* (*DSM*-IV, *DSM*-IV-TR, *DSM*-5, *DSM*-5-TR; American Psychiatric Association, 1994, 2000, 2013, 2022) or International Classification of Diseases (ICD-10 or ICD-11; World Health Organization, 2016, 2019) criteria and/or confirmed by clinical assessment tools (e.g. ADOS; Lord et al., 2012). Studies with self-reports of autism diagnosis without clinical evidence or which measured autistic traits in the general population were excluded. There were no constraints on the age or co-occurring conditions of autistic participants.
2. Ecological momentary assessment: Participants should complete self-report EMA. The EMA should have lasted for at least 3 days and assessed participants’ real-life subjective experience in natural settings repeatedly throughout the study period. We set no restrictions regarding the completion format (electrical devices or paper-and-pencil) and sampling schemes (signal-contingent, interval-contingent or event-contingent) of EMA. Signal-contingent means that signals were randomly/semi-randomly generated over the study sampling hours. Interval-contingent means that participants only respond to questionnaires at pre-determined and equally distributed time points. Event-contingent schemes collect data of a specific event when such an event had taken place (Myin-Germeys & Kuppens, 2022). No restrictions were imposed in terms of the constructs measured by the EMA questionnaires. Studies that only passively collect physiological data by wearable devices were not included.
3. Study characteristics: We included all forms of empirical study that collected data through EMA with autism. Studies using exclusively qualitative data or review analyses were considered as sources, but not included in the current review. Book chapters were excluded.

### Study selection

The screening was conducted in two steps. First, the titles and abstracts of all identified articles were screened according to the inclusion criteria. The reference lists of reviews on related topics were manually scanned for additional articles. Then, the full texts of articles were retrieved and assessed for eligibility. A second reviewer independently screened 20% of randomly selected records from Stage 1 and the inter-rater reliability was assessed by Cohen’s kappa (Cohen, 1960). The study selection process was conducted on Endnote 20 (The EndNote Team, 2013).

### Data extraction

Study information and EMA characteristics were extracted based on a coding scheme adapted from van Roekel et al. (2019). The extracted descriptive information included study details (authors, publication year, country, aims), and participant characteristics (sample size, age, gender, co-occurring conditions, intelligence level and level of autism severity). Descriptive information is presented in tables. The EMA procedures, methodologies, characteristics of questionnaires and challenges/recommendations identified/raised by researchers/participants are summarised in a flowchart and tables. The second reviewer independently extracted data from 20% of the included articles, and inter-rater agreement was calculated (Cohen, 1960).

### Risk of bias

The quality of included EMA studies was assessed following an adapted Strengthening the Reporting of Observational Studies in Epidemiology (STROBE; Von Elm et al., 2007) Checklist for Reporting EMA Studies (CREMAS) developed by Liao et al. (2016). The checklist covers 12 items in the methods and results sections (see details in Liao et al., 2016). We assessed whether each of the included studies has reported these items. A histogram of the total number of reported items and a box plot reflecting the number of studies that reported each specific item is presented.

### Data synthesis

This review synthesised the implementation of EMA in autism studies. First, the research topics and other study/participant characteristics were summarised. Previous research topics were categorised into groups. Second, we used a flowchart to summarise the procedures of included studies. The details of each step and characteristics of EMA procedures were presented in tables, figures and texts. Third, we collected information about the feasibility (i.e. response rate and participants’ qualitative feedback) of EMA. The possible factors that influence the response rate measured by previous studies were summarised. Last, challenges, adaptations in the methodologies of EMA and recommendations raised/identified by researchers and autistic participants were categorised into themes and presented in tables.

### Community involvement

No community partners were involved in this systematic review.

## Results

**Figure 1.**
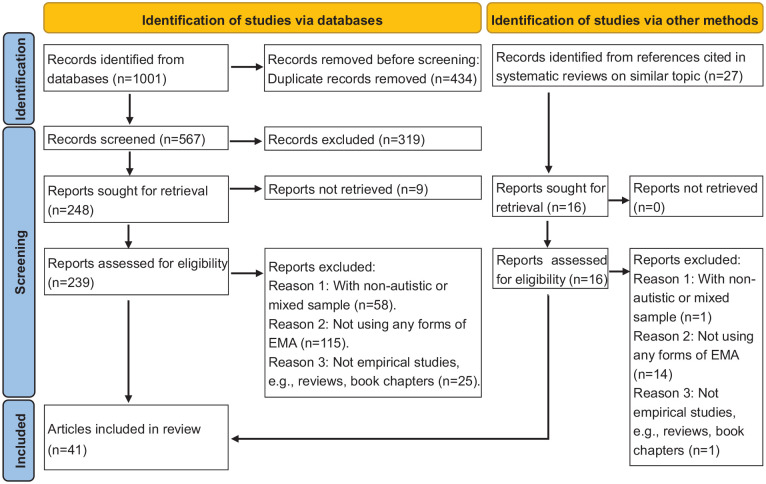
PRISMA flowchart of literature search and article selection.

In total, 1001 records were identified from databases. After removing 434 duplicates manually, 567 articles were further screened and assessed for eligibility. The full text of 239 articles from electronic databases and 16 from reference lists of reviews on related topics were retrieved and assessed for eligibility. Forty-one publications were finally included in the current review. All included publications were published after 1994, with the majority (32 of 41, 78%) published from 2015 onwards. Some of these publications reported EMA datasets from the same sample and so were combined, resulting in 32 unique studies in the current review. The PRISMA flowchart of the study selection process is presented in Figure 1 (Page et al., 2021). The inter-rater reliability for study selection was κ = 0.85 (Cohen, 1960). The agreement for data extraction was over 98%.

### Objective 1 – summary of the study aims and participant characteristics of autism EMA studies

The study characteristics (Objective 1) are presented in Supplemental Table S1 by studies and summarised in Table 1.

**Table 1.** Summary of study characteristics.

Study characteristics	Number of studies (Total *k* = 32)
Sample size (*N*)	*N* = 930, *M* = 29.1, *SD* = 25.4 MIN = 3, MAX = 117
⩽20	15 (46.9%)
20–40	11 (34.4%)
40–60	3 (9.4%)
⩾60	3 (9.4%)
Age	*M* = 22.0, *SD* = 9.11 (*k* = 29) MIN = 8, MAX = 71
Only include <18	9 (29.1%)
Include children <10	3 (9.4%)
Only include >18	14 (43.8%)
Include >60	7 (21.9%)
Gender^a^	Male: 71.2% Female: 26.3%
Co-occurring conditions	18 studies report (56.3%)
Anxiety	11 (34.4%)
Depression	8 (25.0%)
ADHD	9 (28.1%)
Mood/emotional disorder	7 (21.9%)
Intelligence/language requirements	27 studies report requirements (84.4%)
IQ requirements	20 studies report IQ (62.5%), *M* = 107.7, *SD* = 7.0 (*k* = 16) 15 studies specify cut-off IQ scores of >70 5 studies include few participants with IQ <70
Other requirements	Proficient English/French/Chinese language skills (depending on the language used in the country of data collection) Sufficient reading comprehension. Without special education supports for academic or cognitive difficulties in mainstream schools.
Autism traits^b^	18 studies reported autism trait measurements (56.3%)
ADOS^c^	9 (28.1%)
SCQ^d^	8 (25.0%)
ADI^e^	7 (21.9%)
AQ^f^	6 (18.8%)
Research topics	
Affect/emotion	10 (31.3%)
Social experiences	8 (25.0%)
Mental health	7 (21.9%)
Sleep	3 (9.4%)
Feasibility of EMA	4 (1.3%)

a Gender and sex were assumed to be the same for participants in the included primary studies, and thus ‘male’ and ‘female’ are used for discussing gender issues throughout the current review, while we acknowledge that the literature does not currently afford the opportunity to tease apart effects of sex and gender (Lai et al., 2015).

b All studies confirmed that autistic participants scored over the suggested cut-off score for autism.

c Autism Diagnostic Observation Schedule (Lord et al., 2000, 2012).

d Social Communication Questionnaire (Rutter, Bailey, & Lord, 2003).

e Autism Diagnostic Interview (Lord et al., 1994; Rutter, Le Couteur, & Lord, 2003).

f Autism Quotient (Allison et al., 2012; Baron-Cohen et al., 2001).

### Objective 2 – summary of the methodological characteristics and questionnaire designs of autism EMA studies

**Figure 2.**
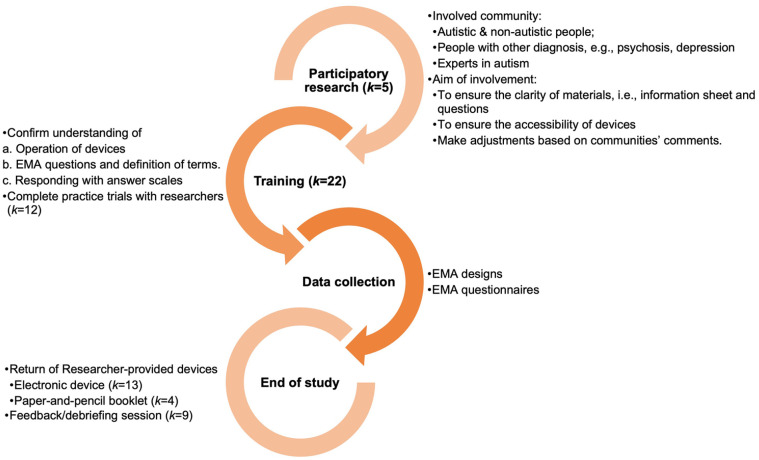
Summary of procedures and methodological characteristics.

A summary of the procedures and methodological characteristics of EMA is presented in Figure 2. This flowchart is a combination of study information from all included studies, so not all the details can be found in each study.

**Figure 3.**
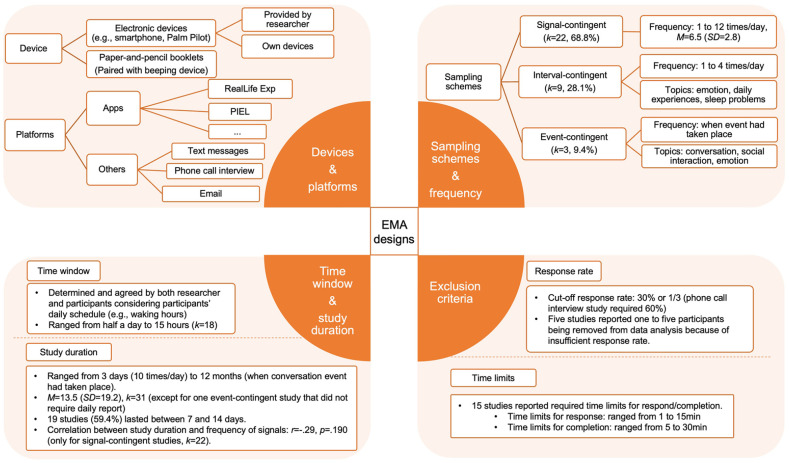
Summary of EMA designs.

The details of EMA designs are presented in Supplemental Table S2 by studies and summarised in Figure 3. Overall, all three common sampling schemes of EMA were found in the included studies. The most widely used approach was the signal-contingent scheme. To avoid some extreme cases in which signals could be distributed very unequally (e.g. all signals are generated in the morning), resulting in underrepresentation of the entire day (Myin-Germeys & Kuppens, 2022), and to minimise disrupting school schedules for the school-aged autistic participants (e.g. Kovac et al., 2016), some studies used a ‘semi-random’ rather than a ‘random’ scheme by setting minimum intervals between signals or dividing the sampling time window into several periods, and thus the signals randomly prompt only within these pre-defined time slots. Studies with random or semi-random signal-contingent schemes are noted in Supplemental Table S2. Six signal-contingent studies instructed participants to respond to signals as much as possible (but can ignore signals when inconvenient or unsafe) and three studies specified responding as soon as possible.

### EMA questionnaires

The development of EMA questionnaires varied across studies. Nine studies adapted EMA questionnaires from previous studies/questionnaires and all studies included questions that were developed specifically for their own study. The types of questions and answering scales in EMA questionnaires are summarised in Table 2. Of the studies that reported the length of questionnaires (27 of 32 studies, 84.4%), the number of questions ranged from 1 to 38 (*M* = 12.2, *SD* = 8.1). Only eight studies stated the mean questionnaire completion time and all of them were less than 5 min.

**Table 2.** Summary of EMA questions and answering scale.

Types of questions	Answering scales	Number of studies (*k*)
Open-ended questions	Total	14
	Typing/writing in text	14
	Drawing	2
	Audio recording	2
Closed-ended questions	Total	26
	Likert rating scale	18
	Multiple/single choice	14
	Tick box	1
	Visual analogue scale	7
	Yes/no	10

### Objective 3 – feasibility of EMA in autism research, as indexed by response rates and autistic participants’ qualitative feedback

The response rates for signal-contingent and interval-contingent studies are summarised in Table 3. The correlation between sampling frequency and response rate among signal-contingent studies (*k* = 21) was not significant (*r* = –0.06, *p* = 0.802). Looking at studies that reported both the questionnaire length and response rate (*k* = 20), a non-significant correlation (*r* = –0.11, *p* = 0.632) was found.

**Table 3.** Summary of response rate.

Sampling schemes	Calculation description	Response rate
Signal-contingent	The number of completed questionnaires out of total questionnaires (*k* = 21)	*M* = 72.1%, *SD* = 13.4 Range = 40%–100%
	Percentage of participants that returned EMA booklets (*k* = 1)	81.5%
Interval-contingent	The number of completed questionnaires out of total questionnaires (*k* = 5)	*M* = 75.1%, *SD* = 20.7 Range = 53.7%–100%
	Percentage of participants that completed EMA questionnaire every sampling day (*k* = 1)	93.3%
	Percentage of participants with EMA data at the end of study (*k* = 2)	20% and 97.2%

Some plausible factors may be associated with response rate. The demographic characteristics, age (Kovac, 2015; Kovac et al., 2016; Song et al., 2023), gender (Khor, Gray, et al., 2014; Khor, Melvin, et al., 2014; Song et al., 2023), and race/ethnicity (Song et al., 2023) were not found to be related to response rate. No group differences were found between autistic and non-autistic participants (Cai et al., 2020; Costache et al., 2024; Rump, 2010; Samson et al., 2015) as well. However, whether IQ influences response rate is more ambiguous: Khor, Gray, et al. (2014) found a positive correlation between IQ and response rate (*r* = 0.46, *p* < 0.01), while Kovac et al. (2016) and Kovac (2015) tested for, but did not find, such a relationship. For studies with longer duration (14 days in Khor, Gray, et al. (2014) and Khor, Melvin, et al. (2014), the response rate decreased with time.

Regarding the qualitative feedback collected at the end of studies, in general, autistic participants gave positive comments for the usage of electronic devices (four studies) and completion of EMA questionnaires (five studies) and reported little disruption to their daily activities (four studies). Although some previous researchers expressed concerns about autistic people’s capacity to transition between activities (Leung & Zakzanis, 2014), autistic participants in Chen et al. (2014) reported that they were able to shift back and forth between responding to signals and daily activities.

### Objective 4 – summary of the implementation challenges, adaptations of EMA methodologies, and recommendations for future autism EMA studies proposed by researchers and autistic participants

Several challenges in the design of EMA were identified by autistic participants and researchers. Following researchers’ previous experience, some made adaptations in the procedures and designs to improve the applicability of EMA for autistic participants. Other problems that have not been addressed were stated by researchers and autistic participants as suggestions for future EMA studies. We summarised these challenges, adaptations and recommendations into several themes/subthemes with descriptions in Tables 4 and 5.

**Table 4.** Challenges of EMA measure.

Themes	Subthemes	Identified by participants^a^	Descriptions
Low response rate	Unaware of signal	✓	Didn’t hear the alert because the beeps were not loud enough in public places, the devices were left at home, etc.
	Inconvenient signalling time	✓	Inappropriate to use the device, for example, during driving, in class, toilet/shower.
	Being frustrated by signals	✓	Signals interrupted daily activity.
		✓	A fixed time window may not fit everyone’s schedule.
		✓	Unpredictability of the beeps and/or waiting for beeps.
		✓	Unreliability of device added to frustration and anxiety, leading to dropouts.
		✓	Beeps were sometimes considered annoying in quiet places.
	Boredom	✓	Questionnaires were too long and feel bored to repeat answering same questions.
Questionnaire designs	Item validity		Questionnaires that were adapted from previous studies may not be previously used in autism, for example, the Positive and Negative Symptom Scale (PANSS) in van der Linden et al. (2020).
			Interpretation of items and options may differ between participants.
	Open-ended responses		Text responses were too ambiguous or not suitable for analysis
		✓	The subjective experiences were in visual, which were hard to be translated/reported in words.
	Closed-ended responses		Responses were limited in the pre-programmed options.
	Missing information		Participants only reported partial experience.
			Experiences outside sampling time window were not collected.
			Missed the opportunity to capture peak affective experiences in the moment of intense affect.
EMA devices	Paper-and-pencil		Cannot ensure ‘in the moment’ completion, the reliability of data is questioned
	Electronic devices	✓	Not user-friendly for everyone. Some people reported difficulties in the usage.
			Possible technical issues could result in loss of data and influence participants’ responds.

a Whether this challenge was reported by participants or not.

**Table 5.** Adaptations of EMA in previous studies and recommendations of EMA for future studies.

Themes	Subthemes	A/R^a^	Descriptions
Improve accessibility of EMA measure	Support understanding	A	Provide training with parents for young autistic participants and autistic participants with cognitive difficulties.
		A	Use easy and straightforward language and structure of tasks to reduce cognitive capacity.
		A^b^	Provide visual supports in information sheets and questionnaires.
	Support responding	A	Open-ended questions: allow for multiple respond media, including drawing^b^ and oral recording.
		A^b^	Closed-ended questions: use simpler response options, for example, dichotomous questions, sliders.
	Sampling time	A&R	Custom time window for autistic participants, for example, only during waking hour.
		R	Participants-initiated trials can reduce frustration caused by interrupting daily activity.
	Sampling time (for school-aged autistic participants)	A	Ask permission from schools and agree on sampling time that would not interfere daily activity but able to capture as much subjective experience as possible.
		A	Collect data outside school time during weekdays/set different time window for weekdays and weekends.
Improve response rate	Optimise electronic devices	R	Allow for controlling volume of signals or setting of vibrate function (and provide relevant trainings).
		R	Ensure the reliability of device, for example, lock devices between signals to ensure privacy and confidentiality of responses.
	Increase motivation	A	Add breaks between sampling days to avoid losing motivation.
		R	Maintain contact with autistic participants to engage them (some autistic participants did not prefer this).^b^
	Response rate for school-aged autistic participants	R	Parents/teachers remind autistic participants to carry device with them and respond to signals.
		R	Inform all teachers, including relief teachers, about students’ participation of study.
		R	Encouragements from parents/teachers/researchers to help maintain autistic participants’ interests.
Improve quantity and quality of gathered EMA data	Quantity of data collection	A	Discuss autistic participants’ responses in follow-up reflective conversations at the end of the study to clarify some ambiguities.
		A	Ask autistic participants to report events/experiences not only at-the-moment, but also since the previous signal.
		R	Extend duration of data collection to gather more comprehensive data on daily experiences.
	Quality of data collection	A	Add breaks between sampling days to check problems in study designs.
		A	Discard first several sets of data since participants need first several signal responses to familiar with the procedure.
		R	Check if the data are self-reported.
		R	Use a multi-modal approach, for example, including physiological measures, to create a more thorough understanding.
Participatory involvement		A&R	Consult with autistic collaborators to gain insight into the utility and protocol of EMA.

a A/R: Adaptation or recommendation. Adaptations refer to designs that are already shown in included EMA studies. Recommendations are suggestions produced by researchers or participants for future studies.

b Suggestions proposed by autistic participants.

### Risk of bias

**Figure 4.**
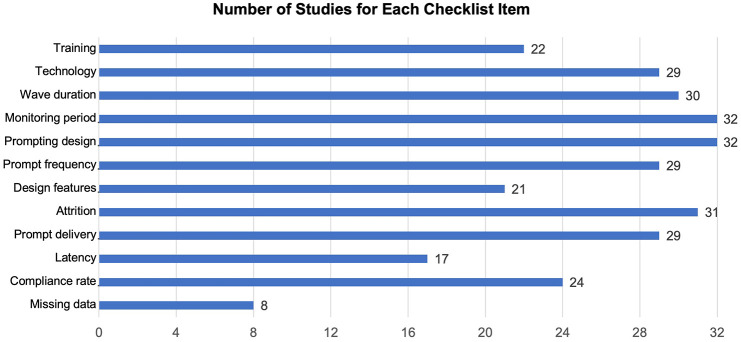
Summary plot of quality assessment results (CREMAS).

Overall, 19 out of 32 studies specified information to allow the coding of more than 10 CREMAS items (12 items in total). Only two studies reported enough information for coding fewer than five items. Over 80% of studies reported most methodological designs, that is, technology (29/32 studies; 91%), sampling scheme (32/32 studies; 100%), frequency (29/32 studies; 91%) and study duration (32/32 studies; 100%). Training was only found in 22 studies (69%). Regarding the ‘design features’ item which refers to designs that address potential biases/problems, 21 (66%) studies specified methods to support participation (e.g. contacting autistic participants during the study or reducing the difficulty of completing questionnaires). The two least-reported items were latency (i.e. the time from signal to response) and missing data (i.e. variables related to response rate). Only 17 (53%) studies stated the time limits for responding/completing of EMA questionnaire. Very few studies (8/32 studies; 25%) conducted further analyses to investigate potential factors associated with response rate. See Figure 4 for the number of studies that reported each item and Supplemental Figures S1 and S2 for further details.

## Discussion

This current review synthesised the literature on EMA with autistic people to understand the feasibility of this method in autism research and to derive ideas for optimising it in future autism studies. Overall, this review demonstrated that EMA is a generally feasible research method for young and middle-aged autistic people with average or above-average intelligence and language ability. Several suggestions to enhance the accessibility and applicability of EMA were co-produced by researchers and autistic participants. Based on these findings, we recommend that autism EMA studies could be optimised by controlling sampling intensity, adapting questionnaire materials, and using incentives to increase engagement.

### Feasibility of EMA in autism research (Objectives 1, 2 & 3)

Across the included studies, the mean EMA response rate with autistic populations (~72%) appears to be acceptable and adequate for EMA data analyses (Chen et al., 2014; Hintzen et al., 2010; Myin-Germeys et al., 2003). However, EMA’s feasibility may vary with autistic people’s demographic and intellectual characteristics. Of the studies in the current review, autism EMA studies were more likely to be carried out with young autistic adults and those in middle age, constraining our ability to draw conclusions about the feasibility of EMA with young children and older people in the autism population. The dearth of autism ageing research was addressed in several previous reviews (e.g. Mason et al., 2022), suggesting that the underrepresentation of older autistic adults is not limited to EMA studies, but rather is characteristic of autism research generally. Similarly, although autistic toddlers and children have been the focus of much research (Jang et al., 2014), collecting and analysing solely self-report information from autistic youth is relatively less common. Several previous studies documented that possible delays in autistic children’s cognitive development may influence autistic children’s abilities to provide an empirically reliable self-report on their subjective experiences (e.g. Ikeda et al., 2014; Mazefsky et al., 2011). Therefore, the underrepresentation of autistic children and older autistic people in EMA studies likely in part reflects a general autism research attention bias, rather than assuming the ‘unfeasibility’ of EMA with youth and elders in the autism population. Furthermore, it is possible, given that EMA studies to date tend to collect data using electronic devices (Myin-Germeys & Kuppens, 2022), that a further obstacle for work with these age groups could be a lack of access to electronic devices throughout the day (e.g. for children of school age) or limited confidence using electronic devices (e.g. for some older adults, see Burke and Naylor (2022)). In two previous EMA reviews, the response rates of EMA with non-autistic youth (76%; Heron et al., 2017) and elders (85%; Yao et al., 2023) were both sufficient and adequate for EMA data analyses. Following these successful implementations of EMA with non-autistic youth and elders, future autism EMA studies could also encompass autistic children and older adults, providing evidence for the feasibility of EMA with autistic people in a wider age range.

Regarding autistic participants’ intellectual characteristics, the underrepresentation of autistic people with ID in EMA studies not only results from selection bias in autism research (e.g. Russell et al., 2019) but also points to some potential challenges in implementing EMA with people with ID. A recent scoping review of EMA (Bakkum et al., 2024) included three studies (Gosens et al., 2024; Hulsmans et al., 2023; Wilson et al., 2020) with people with mild ID or borderline intellectual functioning. Gosens et al. (2024) and Hulsmans et al. (2023) asked participants to complete daily diary (once per day) and both reported average response rates of around 70%. Wilson et al. (2020), which delivered seven EMA questionnaires per sampling day, reported only 34% of compliance rate. Therefore, sampling frequency may influence the likelihood of people with ID responding to EMA questionnaires. Technical difficulties and researchers’ experiences of likely misinterpretation of some gathered information were mentioned by Hulsmans et al. (2023) and Wilson et al. (2020) as well. These potential implementation difficulties suggested important research gaps in the accessibility of EMA for people with ID (Bakkum et al., 2024). As there is a lack of evidence on how well autistic people with ID participate and engage in EMA data collection, whether these feasibility issues are similar in autism EMA studies remains unclear.

### Optimising applicability of EMA in future autism studies (Objective 4)

We summarised three EMA design and implementation suggestions that arose from collaboration between autistic participants and researchers. These were designed to improve the accessibility and applicability of EMA with autistic people. It should be noted that some suggestions are not limited to autism EMA studies but could be applied in research beyond autistic participants and EMA measures.

First, research fatigue comes with the increasing intensity of EMA sampling; therefore, researchers could consider adapting the data collection schedule to reduce the participation burden. In a recent meta-analysis of 477 EMA articles, the number of EMA assessments per sampling day was found to be negatively associated with response rates (Wrzus & Neubauer, 2023). Although this negative relationship across the included studies in the current review was not statistically significant, this preliminary evidence suggests that too-frequent sampling may reduce autistic people’s willingness to continue completing EMA assessments over the study period. Alternatively, the study schedule could be adapted to reduce participant burden by, for example, arranging short intervals between study sessions where no data collection occurs. In one included study, Chen et al. (2015) designed a 2- to 3-day break between two study sessions and stated that this short break helps to prevent autistic participants from losing motivation to complete the EMA surveys. Such ‘measurement burst’ design, with multiple intensive data collection sessions being repeated longitudinally with breaks between them, reduces participant burden for completing the whole EMA. Although Wrzus and Neubauer (2023) did not find a significant difference in response rate and dropout rate between studies with and without implemented break days, the effect of a burst design on feasibility of EMA has not yet been specifically examined across autism studies and requires further investigation.

Second, the number and difficulty of questions in EMA questionnaires could be adapted to make EMA research more accessible for not only autistic participants but also people with diverse levels of cognitive ability. Starting from the length of the EMA questionnaire, in one included study (Chen et al., 2015), autistic children reported that repetitively completing long questionnaires (22 items) is boring. Similarly, Eisele et al. (2022) found that a long questionnaire (60 items) was associated with higher reported momentary burden and lower response rate than a short version (30 items). However, Hasselhorn et al. (2022) did not find evidence for such an effect and suggested that the measured constructs and the time limits for completing EMA questionnaire may possibly influence how the questionnaire length is associated with research burden and response rate. Thus, while ensuring gathering sufficient data for analyses, researchers should consider how to control the number of items in each questionnaire. One option is to use a branching approach in which the number of following questions depends on answers to previous conditionally branched questions. As adopted in several studies included in this review (e.g. Feller et al., 2023, 2024; van Oosterhout et al., 2022), this method can reduce unnecessary questions in certain sampling moments. Regarding the wording of EMA questions, expert consensus suggests the importance of brevity considering participation experience (Eisele et al., 2024).

To make self-report EMA questions more accessible, researchers could consider reducing the difficulty of understanding and responding to questions. For example, a 5-point Likert-type scale with anchors of *very slightly* and *extremely* may be difficult to understand for some autistic children. Across included studies, this response scale could be modified by (1) reducing the language complexity, for example, Kovac et al. (2016) changed the anchor to ‘not at all’ and ‘a lot’, and (2) changing to a simpler dichotomous scale (Chen et al., 2015). Furthermore, graphical representations were used in several included studies. As reported by some autistic participants in Hare et al. (2015), their thoughts may be visual, while EMA questionnaires are mostly based only on words. This mismatch in the format requires autistic people to translate their visual thoughts into words before answering EMA questions. Across the reviewed studies, Kovac et al. (2016) and A. L. Jordan et al. (2021) used graphs to help autistic participants visualise the differences between scales and identify emotions. It is worth noting that using graphical aids in children’s self-reports of moods and experiences has long been used in research (Heron et al., 2017), reflecting its potential to improve the accessibility of research to both autistic and non-autistic populations. However, a general guide for the design of response scales (the overall scope, use of anchors, etc.) is currently unavailable and needs more empirical research (Eisele et al., 2024). Open-ended questions could take more time and effort to complete while providing more insights regarding the research question (van Roekel et al., 2019). Therefore, in addition to the traditional written format of open-ended questions, drawing could be another medium for autistic participants to describe their thoughts and experiences (Chen et al., 2014; Hare et al., 2015).

Last, although not explicitly summarised in the current review, a few included studies briefly described how they maintained and enhanced autistic participants’ engagement throughout the EMA study. Providing direct monetary incentive, as reported in one third of the reviewed studies in Wrzus and Neubauer (2023), was found to be associated with higher response rate, irrespective of the monetary amount. While many incentive strategies were commonly reported and used in EMA studies with non-autistic populations (course credit, lottery, etc., see Wrzus and Neubauer (2023)), their implementation may not be directly applied to autistic participants. For example, autistic people across the lifespan are more likely to show greater intensity regarding special/restricted interests than non-autistic populations (C. J. Jordan & Caldwell-Harris, 2012; Joseph et al., 2013), and thus taking their specific interests into consideration is important when incentivising autistic participants. In Chen et al. (2015), the iPod touch used for EMA data collection was reported as a powerful incentive to keep autistic youth motivated over the study period. Therefore, to maximise the efficacy of incentives, autistic participants’ characteristics, such as their preferred rewards and specific interests, deserve more consideration when designing strategies to enhance autistic participants’ engagement in EMA data collection.

### Future directions

The current review identified several areas that future EMA autism studies could continue to investigate. First, although the current review discussed several factors that possibly influence the feasibility and applicability of EMA, there is a lack of sufficient statistical data reflecting the strength of influence. Studies comparing the quantity and quality of data between EMA with and without, for example, graphical representations in response scales, will provide stronger evidence for designing future autism EMA studies. Second, by modifying the EMA questionnaires and sampling schedule, many other autism areas could be explored by EMA as well. For example, autistic people’s sensory experiences are likely to be influenced by real-time contexts and to fluctuate over time, which cannot be fully captured by retrospective sensory measurements. Incorporating EMA methods with traditional assessment tools could extend our understanding of autistic people’s atypical sensory responses.

## Conclusion

Overall, this review focused on the implementation of EMA with the autistic population. Although both autistic participants and researchers have identified several challenges in the participation and design of EMA, this method was generally acceptable to young and mid-aged autistic people with average or above-average intelligence and language ability, and the response rates were sufficient for researchers to conduct data analyses and make conclusions. It is notable that there is a growing interest in the EMA method in autism research. This review encourages a wider application of EMA in future autism research.

## Supplemental Material

sj-docx-1-aut-10.1177_13623613241305722 – Supplemental material for A systematic review of ecological momentary assessment in autism research Supplemental material, sj-docx-1-aut-10.1177_13623613241305722 for A systematic review of ecological momentary assessment in autism research by Yixin Chen, Zhenyang Xi, Talya Greene and Will Mandy in Autism

sj-docx-2-aut-10.1177_13623613241305722 – Supplemental material for A systematic review of ecological momentary assessment in autism research Supplemental material, sj-docx-2-aut-10.1177_13623613241305722 for A systematic review of ecological momentary assessment in autism research by Yixin Chen, Zhenyang Xi, Talya Greene and Will Mandy in Autism

sj-docx-3-aut-10.1177_13623613241305722 – Supplemental material for A systematic review of ecological momentary assessment in autism research Supplemental material, sj-docx-3-aut-10.1177_13623613241305722 for A systematic review of ecological momentary assessment in autism research by Yixin Chen, Zhenyang Xi, Talya Greene and Will Mandy in Autism

sj-jpg-4-aut-10.1177_13623613241305722 – Supplemental material for A systematic review of ecological momentary assessment in autism research Supplemental material, sj-jpg-4-aut-10.1177_13623613241305722 for A systematic review of ecological momentary assessment in autism research by Yixin Chen, Zhenyang Xi, Talya Greene and Will Mandy in Autism

sj-jpg-5-aut-10.1177_13623613241305722 – Supplemental material for A systematic review of ecological momentary assessment in autism research Supplemental material, sj-jpg-5-aut-10.1177_13623613241305722 for A systematic review of ecological momentary assessment in autism research by Yixin Chen, Zhenyang Xi, Talya Greene and Will Mandy in Autism
