# Supplementary material for: A systematic review of ecological momentary assessment in autism research
Source: Autism. 2024 Dec 18;29(6):1374–89. doi: 10.1177/13623613241305722 (PMC12089685; doi:10.1177/13623613241305722)
Supplement: sj-docx-2-aut-10.1177_13623613241305722 – Supplemental material for A systematic review of ecological momentary assessment in autism research [file sj-docx-2-aut-10.1177_13623613241305722.docx]

**Table S1**

*Overview of included studies*

| Author (year) | Country | Aim* | | Sample size | Gender (M/F) | Age range (*M*,*SD*) | Co-occurring conditions | | Intelligence level | Autism traits |  |
| --- | --- | --- | --- | --- | --- | --- | --- | --- | --- | --- | --- |
| Bal et al. (2024), Mournet et al. (2024) | US | 1 | | 14 | 10/4 | (23.1, 6.2) | Not specified | | Verbal intelligent quotient > 70. Reading comprehension skills reach 6^th^ grade or higher | Not specified |  |
| Chen et al. (2024) | China | 2 | | 82 | 74/8 | 10-16  (12.5, 2.1) | ADHD, emotional disorders, OCD | | Attend mainstream classes without special education support. Have passed Chinese subject. | SCQ |  |
| Costache et al. (2024) | France | 1 | | 29 | 13/14 | 18-67  (28.6, 10.9) | Emotion dysregulation, anxiety, depression, bipolar disorder, OCD, eating disorder, ADHD | | >70 | ADOS-2, ADI-R |  |
| Dallman and Bailliard (2024), Dallman et al. (2022) | US | 1&3, occupational participation | | 17 | 17/0 | 11.0-17.0 (14.0, 2.0) | ADHD, depression | | *M*=106.18 (>85) | Not specified |  |
| Feller et al. (2024) | Switzerland & France | Loneliness | | 48 | 26/22 | 12-30  (18.17, 4.7) | Mood disorders, anxiety, OCD, ODD, PTSD, ADHD | | *M*=105.83 (3 participants with IQ<70) | ADOS-2:11.93 (module 3), 13.76 (module 4), SCQ=17.22, ADI-R |  |
| **Table S1** *(continued)* | | | | | | | | | | | |
| Author (year) | Country | Aim* | Sample size | | Gender (M/F) | Age range (M/SD) | Co-occurring conditions | Intelligence level | | Autism traits |  |
| Ilen et al. (2024) | Switzerland & France | 1 | | 39 | 23/16 | 12-29  (18.37, 4.85) | Anxiety, mood disorder, PTSD, OCD, ODD, tic disorder, Tourette syndrome | | *M*=107.05 (1 participant with IQ<70). Reading comprehension equal to 6^th^ grade in France. | SCQ=17.32, ADOS-2, ADI-R |  |
| Shyu et al. (2024) | China | Quality of life | | 117 | 101/16 | 10-16  (12.6, 2.2) | ADHD, emotional disorder, OCD | | Attend mainstream classes without special education support. Have passed Chinese subject. | SCQ=16.1 |  |
| Feller et al. (2023) | Switzerland & France | 2 | | 26 | 15/11 | 12.0-30.0 (18.4, 5.1) | Phobia, anxiety, ADHD, ODD, mood disorder, OCD | | *M*=108.35 (2 participants with IQ<70) | ADOS-2^1^: 11.3 (module 3), 12.5 (module 4); SCQ^2^: 17.12; ADI^3^ |  |
| Lawson et al. (2023) | Australia | 4 | | 8 | 6/2 | 18-70 | Insomnia | | Not specified | Not specified |  |
| Song et al. (2023) | US | Leisure participation | | 40 | 11/19 | 18-61  (31.15, 9.46) | Anxiety, depression, ADHD, PTSD, bipolar disorder, OCD | | 1 participant with IQ<70 | Not specified |  |
| Cooper et al. (2022) | UK | Cognition | | 54 | 32/20 | 18.0-71.0 (33.8, 14.3) | Not specified | | With cognitive ability to read the information sheet, give consent, respond, and complete the measures. | AQ-10^4^: 7.3 |  |
| **Table S1** *(continued)* | | | | | | | | | | | |
| Author (year) | Country | Aim* | Sample size | | Gender (M/F) | Age range (M/SD) | Co-occurring conditions | Intelligence level | | Autism traits |  |
| Silver and Parsons (2022) | UK | Conversation | | 5 | 2/3 | 18.0-42.0 (31.0, 11.0) | Not specified | | Not specified | Not specified |  |
| van der Linden et al. (2020), van der Linden et al. (2021), van Oosterhout et al. (2022) | Netherland | 1&3  Neuroticism Stress | | 50 | 26/24 | 18.0-64.0 (41.1, 12.9) | Depression | | *M*=110.1 (79-147) | ADOS-2^1^ classification: Autism, Autism spectrum |  |
| Jordan et al. (2021) | UK | 1&3 | 8 | | 7/1 | 19.0-51.0 (31.8, 12.7) | Depression, low mood | Not specified | | Not specified |  |
| Lim et al. (2021) | China | 2 | 76 | | 69/7 | 10.0-16.0 (12.7, 2.1) | ADHD, emotional disorders, OCD | Attend mainstream classes without any special education supports for academic or cognitive difficulties.  Passed Grade 3 Chinese subject without any additional educational support. | | SCQ^2^: 16.6 |  |
| Cai et al. (2020) | Australia | 1&3 | 23 | | 16/7 | 17.5-65.4 (31.4, 14.8) | Not specified | >80 | | AQ-Short^5^: 84.8 |  |
| Gerber et al. (2019) | US | 2 | 23 | | 18/5 | 18.0-47.0 (25.2, 6.8) | Not specified | *M*=107.2 (>70) | | AQ^6^: 35.8 ADOS-2^1^: 5.9 |  |
| **Table S1** *(continued)* | | | | | | | | | | | |
| Author (year) | Country | Aim* | Sample size | | Gender (M/F) | Age range (M/SD) | Co-occurring conditions | Intelligence level | | Autism traits |  |
| Baker and Richdale (2017) | Australia | 4 | 36 | | 17/19 | 21.0-44.0 (34.4, 6.5) | Anxiety, mood disorder, depression | *M*=119.81 (>80) | | ADOS-2^1^: 11.59 AQ^6^: 36.11 (8.20) |  |
| Chen et al. (2016), Chen et al. (2017), Chen, Bundy, et al. (2015) | Australia, China | 2 | 30 | | 16/14 | 16.0-45.0 (26.4, 7.7) | ADHD, depression, anxiety, OCD | Sufficient reading comprehension: >85 in the Reading Comprehension Subtest of Woodcock Reading Mastery Test–Third edition for Australia participants and verbal IQ>70 for Taiwan participants | | Not specified |  |
| Chen, Cordier, et al. (2015), Cordier et al. (2016) | Australia | 2&5 | 6 | | 6/0 | 8.0-12.0  (10.7, 1.1) | Anxiety, social problems, phobia, worry | Attend a mainstream school without educational support for cognitive or academic needs. | | CCBRS^7^: 85.7 (autism), 78.5 (Asperger) [70] |  |
| Hare et al. (2016) | UK | 3 | 9 | | 5/4 | 18.0-65.0 | Anxiety, low mood symptoms | *M*=120 (110-131), fluency in English, verbal comprehension equivalent to 9 years or above. | | AQ^6^: 36.78 |  |
| Kovac et al. (2016), Kovac (2015) | US | 1&5 | 19 | | 14/5 | 9.0-19.0  (14.1, 3.3) | Not specified | *M*=102.2 | | ADOS^8^: 13.2; ADI^3^ |  |
| Hare et al. (2015) | UK | 3 | 20 | | 14/6 | 18.0-59.0 (32.2, 12.2) | Anxiety, paranoia | *M*=113.65 (91-128), fluent in English | | Not specified |  |
| **Table S1** *(continued)* | | | | | | | | | | | |
| Author (year) | Country | Aim* | Sample size | | Gender (M/F) | Age range (M/SD) | Co-occurring conditions | Intelligence level | | Autism traits |  |
| Samson et al. (2015) | US | 1 | 31 | | 28/3 | 8.0-20.0  (12.7, 3.3) | Not specified | *M*=104.31 (64-129) | | ADI^3^ |  |
| Chen et al. (2014) | Australia | 2&5 | 4 | | 2/2 | 16.0-32.0 (22.5, 6.8) | Depression, anxiety, ADHD | Sufficient reading comprehension confirmed by a Woodcock Reading Mastery Test–Third edition, no difficulty comprehending or speaking English | | Not specified |  |
| Khor, Gray, et al. (2014), Khor, Melvin, et al. (2014) | Australia | 3&5 Coping and daily hassles | 31 | | 26/5 | 12.0-19.0 (14.5, 1.8) | Not specified | *M*=99.87 (70-130), speak fluent English and possess basic emotional awareness | | SCQ^2^: 15.68; ADI^3^ |  |
| Abdullah (2012) | US | Family relationship | 31 | | 28/3 | 12.0-18.0 (14.5, 1.9) | Not specified | *M*=95.48, fluent in English | | SCQ^2^: 19.16 |  |
| Hintzen et al. (2010) | Netherland | 2 | 8 | | 7/1 | 19.0-43.0 (28.3, 9.1) | Not specified | *M*=117 (100-129) | | Not specified |  |
| Rump (2010) | US | 1 | 19 | | 16/3 | 11.0-17.0 (14.7, 1.9) | Not specified | *M*=106 (87-127) | | ADOS^8^: 14 |  |
| Humphrey and Lewis (2008) | UK | School experience | 20 (9 did daily record) | | Not specified | 11.0-17.0 | Not specified | Not specified | | Not specified |  |
| **Table S1** *(continued)* | | | | | | | | | | | |
| Author (year) | Country | Aim* | Sample size | | Gender (M/F) | Age range (M/SD) | Co-occurring conditions | Intelligence level | | Autism traits |  |
| Øyane and Bjorvatn (2005) | Norway | 4 | 15 (3 self-reported daily record) | | 14/1 | 15.0-25.0 (19.6, -) | Epilepsy (but not epileptic seizures during the study period) | Not specified | | Not specified |  |
| Hurlburt et al. (1994) | Not specified | Inner experience | 3 | | 3/0 | 24.0-34.0 (27.7, 5.5) | Not specified | *M*=100.67 (90-102) | | Not specified |  |

*Note*. Studies consisting at least partly of the same individuals are in same row.

*Aims are categorised into five groups: 1. Affect/emotion; 2. Social experiences; 3. Mental health conditions; 4. Sleeping problems; 5. Feasibility of Ecological momentary assessment. Studies aims that do not fit in all five groups were stated specifically.

^1^Autism Diagnostic Observation Schedule, second version (Lord et al., 2012), ^2^Social Communication Questionnaire (Rutter, Bailey, et al., 2003), ^3^Autism Diagnostic Interview (Lord et al., 1994; Rutter, Le Couteur, et al., 2003), ^4^Autism Quotient-10 (Allison et al., 2012; Baron-Cohen et al., 2001), ^5^Autism Spectrum Quotient (Hoekstra et al., 2011), ^6^Autism Quotient (Baron-Cohen et al., 2001), ^7^Conners Comprehensive Rating Scale (Conners, 2008), ^8^Autism Diagnostic Observation Schedule (Lord et al., 2000).

**References**

Abdullah, M. M. (2012). *Quality of relationships in families of adolescents with and without autism spectrum disorders* [Doctoral dissertation, University of California, Irvine]. ProQuest Dissertations Publishing. <https://www.proquest.com/docview/1074792402/fulltextPDF/97B8FF563F754DCCPQ/1?accountid=14511>

Allison, C., Auyeung, B., & Baron-Cohen, S. (2012). Toward Brief “Red Flags” for Autism Screening: The Short Autism Spectrum Quotient and the Short Quantitative Checklist in 1,000 Cases and 3,000 Controls. *Journal of the American Academy of Child & Adolescent Psychiatry*, *51*(2), 202-212. <https://doi.org/10.1016/j.jaac.2011.11.003>

Baker, E. K., & Richdale, A. L. (2017). Examining the Behavioural Sleep-Wake Rhythm in Adults with Autism Spectrum Disorder and No Comorbid Intellectual Disability. *Journal of Autism and Developmental Disorders*, *47*(4), 1207-1222. <https://doi.org/10.1007/s10803-017-3042-3>

Bal, V. H., Mournet, A. M., Glascock, T., Shinall, J., Gunin, G., Jadav, N., Zhang, H., Brennan, E., Istvan, E., & Kleiman, E. M. (2024). The emotional support plan: Feasibility trials of a brief, telehealth-based mobile intervention to support coping for autistic adults. *Autism: The International Journal of Research & Practice*, *28*(4), 932-944. <https://doi.org/10.1177/13623613231186035>

Baron-Cohen, S., Wheelwright, S., Skinner, R., Martin, J., & Clubley, E. (2001). The autism-spectrum quotient (AQ): Evidence from asperger syndrome/high-functioning autism, malesand females, scientists and mathematicians. *Journal of Autism and Developmental Disorders*, *31*, 5-17. <https://doi.org/10.1023/A:1005653411471>

Cai, R. Y., Richdale, A. L., Dissanayake, C., & Uljarević, M. (2020). How Does Emotion Regulation Strategy Use and Psychological Wellbeing Predict Mood in Adults With and Without Autism Spectrum Disorder? A Naturalistic Assessment. *Journal of Autism and Developmental Disorders*, *50*(5), 1786-1799. <https://doi.org/10.1007/s10803-019-03934-0>

Chen, Y. R., Ng, D. Y., Tseng, M. H., Bundy, A., & Cordier, R. (2024). The impact of coping behaviors on perceived competence and social anxiety in the everyday social engagement of autistic adolescents. *Autism*, *28*(5), 1268-1279. <https://doi.org/10.1177/13623613231196773>

Chen, Y. W., Bundy, A., Cordier, R., Chien, Y. L., & Einfeld, S. (2016). The experience of social participation in everyday contexts among individuals with autism spectrum disorders: An experience sampling study. *Journal of Autism and Developmental Disorders*, *46*(4), 1403-1414. <https://doi.org/10.1007/s10803-015-2682-4>

Chen, Y. W., Bundy, A., Cordier, R., & Einfeld, S. (2014). Feasibility and usability of experience sampling methodology for capturing everyday experiences of individuals with autism spectrum disorders. *Disability & Health Journal*, *7*(3), 361-366. <https://doi.org/10.1016/j.dhjo.2014.04.004>

Chen, Y. W., Bundy, A. C., Cordier, R., Chien, Y. L., & Einfeld, S. L. (2015). Motivation for everyday social participation in cognitively able individuals with autism spectrum disorder. *Neuropsychiatric Disease & Treatment*, *11*, 2699-2709. <https://doi.org/10.2147/NDT.S87844>

Chen, Y. W., Bundy, A. C., Cordier, R., Chien, Y. L., & Einfeld, S. L. (2017). A cross-cultural exploration of the everyday social participation of individuals with autism spectrum disorders in Australia and Taiwan: An experience sampling study. *Autism*, *21*(2), 231-241. <https://doi.org/10.1177/1362361316636756>

Chen, Y. W., Cordier, R., & Brown, N. (2015). A preliminary study on the reliability and validity of using experience sampling method in children with autism spectrum disorders. *Developmental neurorehabilitation*, *18*(6), 383-389. <https://doi.org/10.3109/17518423.2013.855274>

Conners, C. K. (2008). *Conners comprehensive behavior rating scales: Manual*. Toronto: Multi-Health Systems.

Cooper, K., Russell, A., Calley, S., Chen, H., Kramer, J., & Verplanken, B. (2022). Cognitive processes in autism: Repetitive thinking in autistic versus non-autistic adults. *Autism*, *26*(4), 849-858. <https://doi.org/10.1177/13623613211034380>

Cordier, R., Brown, N., Chen, Y. W., Wilkes-Gillan, S., & Falkmer, T. (2016). Piloting the use of experience sampling method to investigate the everyday social experiences of children with Asperger syndrome/high functioning autism. *Developmental neurorehabilitation*, *19*(2), 103-110. <https://doi.org/10.3109/17518423.2014.915244>

Costache, M. E., Gioia, F., Vanello, N., Greco, A., Lefebvre, F., Capobianco, A., Weibel, S., & Weiner, L. (2024). Exploring Emotion Control and Alexithymia in Autistic Adults: An Ecological Momentary Assessment Study. *Journal of Autism & Developmental Disorders*. <https://doi.org/https://dx.doi.org/10.1007/s10803-024-06551-8>

Dallman, A. R., & Bailliard, A. (2024). Subjective experiences of occupational participation in autistic adolescents in the US: A multiple-case study using experience sampling methodology. *Journal of Occupational Science*, *31*(3), 516-529. <https://doi.org/10.1080/14427591.2024.2393163>

Dallman, A. R., Bailliard, A., & Harrop, C. (2022). Identifying Predictors of Momentary Negative Affect and Depression Severity in Adolescents with Autism: An Exploratory Ecological Momentary Assessment Study. *Journal of Autism & Developmental Disorders*, *52*(1), 291-303. <https://doi.org/10.1007/s10803-021-04935-8>

Feller, C., Ilen, L., Eliez, S., & Schneider, M. (2023). Characterizing Daily-Life Social Interactions in Adolescents and Young Adults with Neurodevelopmental Disorders: A Comparison Between Individuals with Autism Spectrum Disorders and 22q11.2 Deletion Syndrome. *Journal of Autism & Developmental Disorders*, *53*(1), 245-262. <https://doi.org/10.1007/s10803-021-05423-9>

Feller, C., Ilen, L., Eliez, S., & Schneider, M. (2024). Loneliness in daily life: A comparison between youths with autism spectrum disorders and 22q11.2 deletion syndrome (22q11DS). *Autism research: Official Journal of the International Society for Autism Research*, *17*(10), 2004-2017. <https://doi.org/10.1002/aur.3173>

Gerber, A. H., Girard, J. M., Scott, S. B., & Lerner, M. D. (2019). Alexithymia - Not autism - is associated with frequency of social interactions in adults. *Behaviour Research & Therapy*, *123*, Article 103477. <https://doi.org/10.1016/j.brat.2019.103477>

Hare, D. J., Gracey, C., & Wood, C. (2016). Anxiety in high-functioning autism: A pilot study of experience sampling using a mobile platform. *Autism*, *20*(6), 730-743. <https://doi.org/10.1177/1362361315604817>

Hare, D. J., Wood, C., Wastell, S., & Skirrow, P. (2015). Anxiety in Asperger's syndrome: Assessment in real time. *Autism*, *19*(5), 542-552. <https://doi.org/10.1177/1362361314531340>

Hintzen, A., Delespaul, P., van Os, J., & Myin-Germeys, I. (2010). Social needs in daily life in adults with Pervasive Developmental Disorders. *Psychiatry Research*, *179*(1), 75-80. <https://doi.org/10.1016/j.psychres.2010.06.014>

Hoekstra, R. A., Vinkhuyzen, A. A., Wheelwright, S., Bartels, M., Boomsma, D. I., Baron-Cohen, S., Posthuma, D., & Van Der Sluis, S. (2011). The construction and validation of an abridged version of the autism-spectrum quotient (AQ-Short). *Journal of Autism and Developmental Disorders*, *41*, 589-596. <https://doi.org/10.1007/s10803-010-1073-0>

Humphrey, N., & Lewis, S. (2008). 'Make me normal': the views and experiences of pupils on the autistic spectrum in mainstream secondary schools. *Autism*, *12*(1), 23-46. <https://doi.org/10.1177/1362361307085267>

Hurlburt, R., Happe, F., & Frith, U. (1994). Sampling the form of inner experience in three adults with Asperger syndrome. *Psychological Medicine*, *24*(2), 385-395. <https://doi.org/10.1017/S0033291700027367>

Ilen, L., Feller, C., & Schneider, M. (2024). Cognitive emotion regulation difficulties increase affective reactivity to daily-life stress in autistic adolescents and young adults. *Autism*, *28*(7), 1703-1718. <https://doi.org/10.1177/13623613231204829>

Jordan, A. L., Marczak, M., & Knibbs, J. (2021). 'I Felt Like I was Floating in Space': Autistic Adults' Experiences of Low Mood and Depression. *Journal of Autism & Developmental Disorders*, *51*(5), 1683-1694. <https://doi.org/10.1007/s10803-020-04638-6>

Khor, A. S., Gray, K. M., Reid, S. C., & Melvin, G. A. (2014). Feasibility and validity of ecological momentary assessment in adolescents with high-functioning autism and Asperger's disorder. *Journal of adolescence*, *37*(1), 37-46. <https://doi.org/10.1016/j.adolescence.2013.10.005>

Khor, A. S., Melvin, G. A., Reid, S. C., & Gray, K. M. (2014). Coping, daily hassles and behavior and emotional problems in adolescents with high-functioning autism/Asperger's disorder. *Journal of Autism and Developmental Disorders*, *44*(3), 593-608. <https://doi.org/10.1007/s10803-013-1912-x>

Kovac, M., Mosner, M., Miller, S., Hanna, E. K., & Dichter, G. S. (2016). Experience sampling of positive affect in adolescents with autism: Feasibility and preliminary findings. *Research in Autism Spectrum Disorders*, *29-30*, 57-65. <https://doi.org/10.1016/j.rasd.2016.06.003>

Kovac, M. L. (2015). *Affect-modulated postauricular reflexes of children with Autism Spectrum Disorder* [Doctoral dissertation, The University of North Carolina at Chapel]. ProQuest Dissertations Publishing. <https://www.proquest.com/openview/65ee40d68a7a2ab39582bae49b2bfb77/1?pq-origsite=gscholar&cbl=18750>

Lawson, L. P., Richdale, A. L., Denney, K., & Morris, E. M. J. (2023). ACT-i, an insomnia intervention for autistic adults: a pilot study. *Behavioural & Cognitive Psychotherapy*, *51*(2), 146-163. <https://doi.org/10.1017/S1352465822000571>

Lim, V. H. T., Chen, Y. R., Tseng, M. H., Bundy, A., & Cordier, R. (2021). The impact of caregiver stigma on real-life social experience of Taiwanese adolescents with autism spectrum disorder. *Autism*, *25*(7), 1859-1871. <https://doi.org/10.1177/13623613211004329>

Lord, C., Risi, S., Lambrecht, L., Cook, E. H., Leventhal, B. L., DiLavore, P. C., Pickles, A., & Rutter, M. (2000). The Autism Diagnostic Observation Schedule—Generic: A standard measure of social and communication deficits associated with the spectrum of autism. *Journal of Autism and Developmental Disorders*, *30*, 205-223. <https://doi.org/10.1023/A:1005592401947>

Lord, C., Rutter, M., DiLavore, P., Risi, S., Gotham, K., & Bishop, S. (2012). *Autism Diagnostic Observation Schedule, Second Edition*. Torrence, CA: Western Psychological Services.

Lord, C., Rutter, M., & Le Couteur, A. (1994). Autism Diagnostic Interview-Revised: a revised version of a diagnostic interview for caregivers of individuals with possible pervasive developmental disorders. *Journal of Autism and Developmental Disorders*, *24*(5), 659-685. <https://doi.org/10.1007/BF02172145>

Mournet, A. M., Gunin, G., Shinall, J., Brennan, E., Jadav, N., Istvan, E., Kleiman, E. M., & Bal, V. H. (2024). The impact of measurement on clinical trials: Comparison of preliminary outcomes of a brief mobile intervention for autistic adults using multiple measurement approaches. *Autism research: Official Journal of the International Society for Autism Research*, *17*(2), 432-442. <https://doi.org/10.1002/aur.3095>

Øyane, N. M. F., & Bjorvatn, B. (2005). Sleep disturbances in adolescents and young adults with autism and Asperger syndrome. *Autism*, *9*(1), 83-94. <https://doi.org/10.1177/1362361305049031>

Rump, K. M. (2010). *Affective experiences in adolescents with autism: An EMA study* [Doctoral dissertation, University of Pittsburgh]. ProQuest Dissertations Publishing. <https://www.proquest.com/docview/890110703?parentSessionId=u7QqvOofb%2FP2A07%2FPXl9xrzWuVOUbUIsWbIB8CwKhgg%3D&pq-origsite=primo&accountid=14511>

Rutter, M., Bailey, A., & Lord, C. (2003). *The Social Communication Questionnaire: Manual*. Los Angeles, CA: Western Psychological Services.

Rutter, M., Le Couteur, A., & Lord, C. (2003). *Autism diagnostic interview-Revised manual*. Los Angeles, CA: Western Psychological Services.

Samson, A. C., Wells, W. M., Phillips, J. M., Hardan, A. Y., & Gross, J. J. (2015). Emotion regulation in autism spectrum disorder: evidence from parent interviews and children's daily diaries. *Journal of Child Psychology and Psychiatry*, *56*(8), 903-913. <https://doi.org/10.1111/jcpp.12370>

Shyu, H. J., Ryan Chen, Y. W., Yih Ng, D., Bundy, A., Tseng, M. H., & Cordier, R. (2024). Does the PedsQL reflect the real-time quality of life in autistic adolescents? A comparison with the experience sampling methodology. *Disability & Health Journal*, Article 101690. <https://doi.org/10.1016/j.dhjo.2024.101690>

Silver, K., & Parsons, S. (2022). Perspectives of autistic adults on the strategies that help or hinder successful conversations. *Autism & Developmental Language Impairments*, *7*, 1-14. <https://doi.org/10.1177/23969415221101113>

Song, W., Zheng, L., Ticha, R., Abery, B., & Nguyen-Feng, V. N. (2023). Leisure Participation of Autistic Adults: An Ecological Momentary Assessment Feasibility Study. *American Journal on Intellectual & Developmental Disabilities*, *128*(4), 319-333. <https://doi.org/10.1352/1944-7558-128.4.319>

van der Linden, K., Simons, C., van Amelsvoort, T., & Marcelis, M. (2020). Lifetime and Momentary Psychotic Experiences in Adult Males and Females With an Autism Spectrum Disorder. *Frontiers in Psychiatry*, *11*, Article 766. <https://doi.org/https://dx.doi.org/10.3389/fpsyt.2020.00766>

van der Linden, K., Simons, C., Viechtbauer, W., Ottenheijm, E., van Amelsvoort, T., & Marcelis, M. (2021). A momentary assessment study on emotional and biological stress in adult males and females with autism spectrum disorder. *Scientific Reports*, *11*, Article 14160. <https://doi.org/https://dx.doi.org/10.1038/s41598-021-93159-y>

van Oosterhout, J., van der Linden, K., Simons, C. J. P., van Amelsvoort, T., & Marcelis, M. (2022). Exploring the autism spectrum: Moderating effects of neuroticism on stress reactivity and on the association between social context and negative affect. *Development & Psychopathology*, *34*(4), 1366-1375. <https://doi.org/10.1017/S0954579420002278>
