# Supplementary material for: A systematic review of ecological momentary assessment in autism research
Source: Autism. 2024 Dec 18;29(6):1374–89. doi: 10.1177/13623613241305722 (PMC12089685; doi:10.1177/13623613241305722)
Supplement: sj-docx-3-aut-10.1177_13623613241305722 – Supplemental material for A systematic review of ecological momentary assessment in autism research [file sj-docx-3-aut-10.1177_13623613241305722.docx]

| **Table S2**  *EMA characteristics of included studies* | | | | | | | | |
| --- | --- | --- | --- | --- | --- | --- | --- | --- |
| Author (year) | Training | Device +  platform | Sampling scheme | Signals/day | Study duration (day) | Number of questions | Answering scales | Response rate^*^ (%) |
| Bal et al. (2024), Mournet et al. (2024) | Not specified | Own smartphone + Metric Wire | Signal-contingent^1^ | 4 | 6 or 7 weeks | 13 | Not specified | 40.0 |
| Chen et al. (2024) | Yes | RP-iPhone | Signal-contingent^1^ | 7 | 7 | 4 | Likert scale | 74.1 |
| Costache et al. (2024) | Yes | Own smartphone + online questionnaire (text message) | Signal/event-contingent^1^ | 12 | 7 | 6 | Multiple-choice, visual analogue scale | 92.7 |
| Dallman and Bailliard (2024), Dallman et al. (2022) | Yes | Own smartphone + online questionnaire (text message) | Signal-contingent^1^ | 6 | 7 | 15 | Likert scale, text | 75.1 |
| Feller et al. (2024) | Yes | Own smartphone + RealLife Exp App | Signal-contingent^1^ | 8 | 6 | 33 to 38 | Likert scale, yes/no | 70.2 |
| Ilen et al. (2024) | Not specified | Own smartphone + RealLife Exp App | Signal-contingent^1^ | 8 | 6 | 18 | Likert scale, yes/no | 68 |
| Shyu et al. (2024) | Yes | RP-iPhone + PIEL App | Signal-contingent^1^ | 7 | 7 | 7 | Likert scale, yes/no | 76.2 |
| Feller et al. (2023) | Yes | Own smartphone + RealLife Exp App | Signal-contingent^1^ | 8 | 6 | 12 to 14 | Likert scale, multiple-choice | 70.5 |
| **Table S2** *(continued)* | | | | | | | | |
| Author (year) | Training | Device +  platform | Sampling scheme | Signals/day | Study duration (day) | Number of questions | Answering scales | Response rate^*^ (%) |
| Lawson et al. (2023) | Yes | Online diary | Interval-contingent | 1 | 105 | 4 | Not specified | Not specified |
| Song et al. (2023) | Yes | Own smartphone + online questionnaire (text message) | Interval-contingent | 1 | 30 | 4 | Multiple-choice, text, rating | 90.2 |
| Cooper et al. (2022) | Yes | Paper booklets & alarms | Signal-contingent^2^ | 5 | 5 | 3 | Text/drawing, tick box, visual analogue scale | Not specified |
| Silver and Parsons (2022) | Not specified | Not specified | Event-contingent | NA^a^ | 4-12 months | Not specified | Not specified | Not specified |
| van der Linden et al. (2020), van der Linden et al. (2021), van Oosterhout et al. (2022) | Not specified | RP-iPod/own smartphone + PsyMate App | Signal-contingent^2^ | 10 | 10 | 16 to 17 | Likert scales, multiple-choice | 79.8 |
| Jordan et al. (2021) | Not specified | Not specified | Interval-contingent | 1 | 7 | Not specified | Not specified | Not specified |
| Lim et al. (2021) | Yes | RP-iPhone + PIEL App | Signal-contingent^2^ | 7** | 7 | 6 | Multiple-choice, yes/no, visual analogue scale | 73.4 |
| Cai et al. (2020) | Yes | RP-iPad mini + PIEL App | Interval-contingent | 3 | 5 to 10 | 7 to 28 | Likert scale, multiple-choice, yes/no, text | 77.0 |
| **Table S2** *(continued)* | | | | | | | | |
| Author (year) | Training | Device +  platform | Sampling scheme | Signals/day | Study duration (day) | Number of questions | Answering scales | Response rate^*^ (%) |
| Gerber et al. (2019) | Yes | Own smartphone + Google Form survey (text message) | Signal/event-contingent^1^ | 12 | 7 | 1 | yes/no | 71.8 |
| Baker and Richdale (2017) | Not specified | Online diary | Interval-contingent | 2 | 14 | 5 | Text | Not specified |
| Chen et al. (2016), Chen et al. (2017), Chen, Bundy, et al. (2015) | Yes | RP-iPod Touch/own iOS device + PIEL App | Signal-contingent^2^ | 7 | 7 | 3 to 8 | Multiple-choice, yes/no, Likert scale | 77.6 |
| Chen, Cordier, et al. (2015), Cordier et al. (2016) | Yes | RP-iPod Touch + PIEL App | Signal-contingent^1^ | 7 | 7 | 22 | Visual analogue scale, yes/no, multiple-choice | 56.8 |
| Hare et al. (2016) | Yes | RP-Palm Pilot + iESP software | Signal-contingent^1^ | 10 | 6 | 13 | Text, multiple choice, analogue scale | 60.6 |
| Kovac et al. (2016), Kovac (2015) | Yes | Own smartphone/ computer + Qualtrics survey (email) | Signal-contingent^1^ | 1 (weekdays) or 2 (weekends) | 4 | 18 | Text, Likert scale | 85.0 |
| **Table S2** *(continued)* | | | | | | | | |
| Author (year) | Training | Device +  platform | Sampling scheme | Signals/day | Study duration (day) | Number of questions | Answering scales | Response rate^*^ (%) |
| Hare et al. (2015) | Not specified | RP-Palm Pilot + Palm Pilot Questionnaire | Signal-contingent^2^ | 10 | 3 | 13 | Text, multiple choice, visual analogue | 50.8 |
| Samson et al. (2015) | Not specified | Email web link to daily diary | Interval-contingent | 1 | 10 | 4 | Likert scale | Not specified |
| Chen et al. (2014) | Yes | Own/RP-iPad Touch/iPhone + PIEL App | Signal-contingent^2^ | 7 | 7 | 20 | Multiple-choice, yes/no, visual analog scales | 71.0 |
| Khor, Gray, et al. (2014), Khor, Melvin, et al. (2014) | Yes | RP-Sony mobile phone + mobiletype programme | Signal-contingent^1^ | 4** | 14 | 9 to 10 | Likert scale, multiple choice, text | 61.8 |
| Abdullah (2012) | Yes | RP-Palm Centro smartphone + pre-programmed questionnaire | Interval-contingent | 1 | 7 | Not specified | Likert scale | Not specified |
| Hintzen et al. (2010) | Yes | Paper booklet & digital wristwatch | Signal-contingent^2^ | 10 | 6 | 13 | Likert scale, yes/no, choice | 80.0 |
| Rump (2010) | Yes | RP-cellular phone | Signal-contingent^1^ | 2 (weekdays) or 5 (weekends) | 14 | 25 | Interview | 78.0 |
| **Table S2** *(continued)* | | | | | | | | |
| Author (year) | Training | Device +  platform | Sampling scheme | Signals/day | Study duration (day) | Number of questions | Answering scales | Response rate^*^ (%) |
| Humphrey and Lewis (2008) | Not specified | Paper booklet /PC/dictaphone | Interval-contingent | Not specified | 30 | Not specified | Text | Not specified |
| Øyane and Bjorvatn (2005) | Not specified | Not specified | Interval-contingent | Not specified | 14 | 10 | Likert scale | Not specified |
| Hurlburt et al. (1994) | Yes | Paper booklet & beeping device | Signal-contingent^2^ | 1 to 6 | 5 | Not specified | Text | 100.0 |

RP = Research group provided

* Response rate = the number of completed questionnaires out of total questionnaires. Response rate being calculated by other methods are noted as ‘not specified’ in the table.

** Different time window between weekdays and weekends.

^a^ When events had taken place.

^1^ Semi-random signals

^2^ Random signals

**References**

Abdullah, M. M. (2012). *Quality of relationships in families of adolescents with and without autism spectrum disorders* [Doctoral dissertation, University of California, Irvine]. ProQuest Dissertations Publishing. <https://www.proquest.com/docview/1074792402/fulltextPDF/97B8FF563F754DCCPQ/1?accountid=14511>

Baker, E. K., & Richdale, A. L. (2017). Examining the Behavioural Sleep-Wake Rhythm in Adults with Autism Spectrum Disorder and No Comorbid Intellectual Disability. *Journal of Autism and Developmental Disorders*, *47*(4), 1207-1222. <https://doi.org/10.1007/s10803-017-3042-3>

Bal, V. H., Mournet, A. M., Glascock, T., Shinall, J., Gunin, G., Jadav, N., Zhang, H., Brennan, E., Istvan, E., & Kleiman, E. M. (2024). The emotional support plan: Feasibility trials of a brief, telehealth-based mobile intervention to support coping for autistic adults. *Autism: The International Journal of Research & Practice*, *28*(4), 932-944. <https://doi.org/10.1177/13623613231186035>

Cai, R. Y., Richdale, A. L., Dissanayake, C., & Uljarević, M. (2020). How Does Emotion Regulation Strategy Use and Psychological Wellbeing Predict Mood in Adults With and Without Autism Spectrum Disorder? A Naturalistic Assessment. *Journal of Autism and Developmental Disorders*, *50*(5), 1786-1799. <https://doi.org/10.1007/s10803-019-03934-0>

Chen, Y. R., Ng, D. Y., Tseng, M. H., Bundy, A., & Cordier, R. (2024). The impact of coping behaviors on perceived competence and social anxiety in the everyday social engagement of autistic adolescents. *Autism*, *28*(5), 1268-1279. <https://doi.org/10.1177/13623613231196773>

Chen, Y. W., Bundy, A., Cordier, R., Chien, Y. L., & Einfeld, S. (2016). The experience of social participation in everyday contexts among individuals with autism spectrum disorders: An experience sampling study. *Journal of Autism and Developmental Disorders*, *46*(4), 1403-1414. <https://doi.org/10.1007/s10803-015-2682-4>

Chen, Y. W., Bundy, A., Cordier, R., & Einfeld, S. (2014). Feasibility and usability of experience sampling methodology for capturing everyday experiences of individuals with autism spectrum disorders. *Disability & Health Journal*, *7*(3), 361-366. <https://doi.org/10.1016/j.dhjo.2014.04.004>

Chen, Y. W., Bundy, A. C., Cordier, R., Chien, Y. L., & Einfeld, S. L. (2015). Motivation for everyday social participation in cognitively able individuals with autism spectrum disorder. *Neuropsychiatric Disease & Treatment*, *11*, 2699-2709. <https://doi.org/10.2147/NDT.S87844>

Chen, Y. W., Bundy, A. C., Cordier, R., Chien, Y. L., & Einfeld, S. L. (2017). A cross-cultural exploration of the everyday social participation of individuals with autism spectrum disorders in Australia and Taiwan: An experience sampling study. *Autism*, *21*(2), 231-241. <https://doi.org/10.1177/1362361316636756>

Chen, Y. W., Cordier, R., & Brown, N. (2015). A preliminary study on the reliability and validity of using experience sampling method in children with autism spectrum disorders. *Developmental neurorehabilitation*, *18*(6), 383-389. <https://doi.org/10.3109/17518423.2013.855274>

Cooper, K., Russell, A., Calley, S., Chen, H., Kramer, J., & Verplanken, B. (2022). Cognitive processes in autism: Repetitive thinking in autistic versus non-autistic adults. *Autism*, *26*(4), 849-858. <https://doi.org/10.1177/13623613211034380>

Cordier, R., Brown, N., Chen, Y. W., Wilkes-Gillan, S., & Falkmer, T. (2016). Piloting the use of experience sampling method to investigate the everyday social experiences of children with Asperger syndrome/high functioning autism. *Developmental neurorehabilitation*, *19*(2), 103-110. <https://doi.org/10.3109/17518423.2014.915244>

Costache, M. E., Gioia, F., Vanello, N., Greco, A., Lefebvre, F., Capobianco, A., Weibel, S., & Weiner, L. (2024). Exploring Emotion Control and Alexithymia in Autistic Adults: An Ecological Momentary Assessment Study. *Journal of Autism & Developmental Disorders*. <https://doi.org/https://dx.doi.org/10.1007/s10803-024-06551-8>

Dallman, A. R., & Bailliard, A. (2024). Subjective experiences of occupational participation in autistic adolescents in the US: A multiple-case study using experience sampling methodology. *Journal of Occupational Science*, *31*(3), 516-529. <https://doi.org/10.1080/14427591.2024.2393163>

Dallman, A. R., Bailliard, A., & Harrop, C. (2022). Identifying Predictors of Momentary Negative Affect and Depression Severity in Adolescents with Autism: An Exploratory Ecological Momentary Assessment Study. *Journal of Autism & Developmental Disorders*, *52*(1), 291-303. <https://doi.org/10.1007/s10803-021-04935-8>

Feller, C., Ilen, L., Eliez, S., & Schneider, M. (2023). Characterizing Daily-Life Social Interactions in Adolescents and Young Adults with Neurodevelopmental Disorders: A Comparison Between Individuals with Autism Spectrum Disorders and 22q11.2 Deletion Syndrome. *Journal of Autism & Developmental Disorders*, *53*(1), 245-262. <https://doi.org/10.1007/s10803-021-05423-9>

Feller, C., Ilen, L., Eliez, S., & Schneider, M. (2024). Loneliness in daily life: A comparison between youths with autism spectrum disorders and 22q11.2 deletion syndrome (22q11DS). *Autism research: Official Journal of the International Society for Autism Research*, *17*(10), 2004-2017. <https://doi.org/10.1002/aur.3173>

Gerber, A. H., Girard, J. M., Scott, S. B., & Lerner, M. D. (2019). Alexithymia - Not autism - is associated with frequency of social interactions in adults. *Behaviour Research & Therapy*, *123*, Article 103477. <https://doi.org/10.1016/j.brat.2019.103477>

Hare, D. J., Gracey, C., & Wood, C. (2016). Anxiety in high-functioning autism: A pilot study of experience sampling using a mobile platform. *Autism*, *20*(6), 730-743. <https://doi.org/10.1177/1362361315604817>

Hare, D. J., Wood, C., Wastell, S., & Skirrow, P. (2015). Anxiety in Asperger's syndrome: Assessment in real time. *Autism*, *19*(5), 542-552. <https://doi.org/10.1177/1362361314531340>

Hintzen, A., Delespaul, P., van Os, J., & Myin-Germeys, I. (2010). Social needs in daily life in adults with Pervasive Developmental Disorders. *Psychiatry Research*, *179*(1), 75-80. <https://doi.org/10.1016/j.psychres.2010.06.014>

Humphrey, N., & Lewis, S. (2008). 'Make me normal': the views and experiences of pupils on the autistic spectrum in mainstream secondary schools. *Autism*, *12*(1), 23-46. <https://doi.org/10.1177/1362361307085267>

Hurlburt, R., Happe, F., & Frith, U. (1994). Sampling the form of inner experience in three adults with Asperger syndrome. *Psychological Medicine*, *24*(2), 385-395. <https://doi.org/10.1017/S0033291700027367>

Ilen, L., Feller, C., & Schneider, M. (2024). Cognitive emotion regulation difficulties increase affective reactivity to daily-life stress in autistic adolescents and young adults. *Autism*, *28*(7), 1703-1718. <https://doi.org/10.1177/13623613231204829>

Jordan, A. L., Marczak, M., & Knibbs, J. (2021). 'I Felt Like I was Floating in Space': Autistic Adults' Experiences of Low Mood and Depression. *Journal of Autism & Developmental Disorders*, *51*(5), 1683-1694. <https://doi.org/10.1007/s10803-020-04638-6>

Khor, A. S., Gray, K. M., Reid, S. C., & Melvin, G. A. (2014). Feasibility and validity of ecological momentary assessment in adolescents with high-functioning autism and Asperger's disorder. *Journal of adolescence*, *37*(1), 37-46. <https://doi.org/10.1016/j.adolescence.2013.10.005>

Khor, A. S., Melvin, G. A., Reid, S. C., & Gray, K. M. (2014). Coping, daily hassles and behavior and emotional problems in adolescents with high-functioning autism/Asperger's disorder. *Journal of Autism and Developmental Disorders*, *44*(3), 593-608. <https://doi.org/10.1007/s10803-013-1912-x>

Kovac, M., Mosner, M., Miller, S., Hanna, E. K., & Dichter, G. S. (2016). Experience sampling of positive affect in adolescents with autism: Feasibility and preliminary findings. *Research in Autism Spectrum Disorders*, *29-30*, 57-65. <https://doi.org/10.1016/j.rasd.2016.06.003>

Kovac, M. L. (2015). *Affect-modulated postauricular reflexes of children with Autism Spectrum Disorder* [Doctoral dissertation, The University of North Carolina at Chapel]. ProQuest Dissertations Publishing. <https://www.proquest.com/openview/65ee40d68a7a2ab39582bae49b2bfb77/1?pq-origsite=gscholar&cbl=18750>

Lawson, L. P., Richdale, A. L., Denney, K., & Morris, E. M. J. (2023). ACT-i, an insomnia intervention for autistic adults: a pilot study. *Behavioural & Cognitive Psychotherapy*, *51*(2), 146-163. <https://doi.org/10.1017/S1352465822000571>

Lim, V. H. T., Chen, Y. R., Tseng, M. H., Bundy, A., & Cordier, R. (2021). The impact of caregiver stigma on real-life social experience of Taiwanese adolescents with autism spectrum disorder. *Autism*, *25*(7), 1859-1871. <https://doi.org/10.1177/13623613211004329>

Mournet, A. M., Gunin, G., Shinall, J., Brennan, E., Jadav, N., Istvan, E., Kleiman, E. M., & Bal, V. H. (2024). The impact of measurement on clinical trials: Comparison of preliminary outcomes of a brief mobile intervention for autistic adults using multiple measurement approaches. *Autism research: Official Journal of the International Society for Autism Research*, *17*(2), 432-442. <https://doi.org/10.1002/aur.3095>

Øyane, N. M. F., & Bjorvatn, B. (2005). Sleep disturbances in adolescents and young adults with autism and Asperger syndrome. *Autism*, *9*(1), 83-94. <https://doi.org/10.1177/1362361305049031>

Rump, K. M. (2010). *Affective experiences in adolescents with autism: An EMA study* [Doctoral dissertation, University of Pittsburgh]. ProQuest Dissertations Publishing. <https://www.proquest.com/docview/890110703?parentSessionId=u7QqvOofb%2FP2A07%2FPXl9xrzWuVOUbUIsWbIB8CwKhgg%3D&pq-origsite=primo&accountid=14511>

Samson, A. C., Wells, W. M., Phillips, J. M., Hardan, A. Y., & Gross, J. J. (2015). Emotion regulation in autism spectrum disorder: evidence from parent interviews and children's daily diaries. *Journal of Child Psychology and Psychiatry*, *56*(8), 903-913. <https://doi.org/10.1111/jcpp.12370>

Shyu, H. J., Ryan Chen, Y. W., Yih Ng, D., Bundy, A., Tseng, M. H., & Cordier, R. (2024). Does the PedsQL reflect the real-time quality of life in autistic adolescents? A comparison with the experience sampling methodology. *Disability & Health Journal*, Article 101690. <https://doi.org/10.1016/j.dhjo.2024.101690>

Silver, K., & Parsons, S. (2022). Perspectives of autistic adults on the strategies that help or hinder successful conversations. *Autism & Developmental Language Impairments*, *7*, 1-14. <https://doi.org/10.1177/23969415221101113>

Song, W., Zheng, L., Ticha, R., Abery, B., & Nguyen-Feng, V. N. (2023). Leisure Participation of Autistic Adults: An Ecological Momentary Assessment Feasibility Study. *American Journal on Intellectual & Developmental Disabilities*, *128*(4), 319-333. <https://doi.org/10.1352/1944-7558-128.4.319>

van der Linden, K., Simons, C., van Amelsvoort, T., & Marcelis, M. (2020). Lifetime and Momentary Psychotic Experiences in Adult Males and Females With an Autism Spectrum Disorder. *Frontiers in Psychiatry*, *11*, Article 766. <https://doi.org/https://dx.doi.org/10.3389/fpsyt.2020.00766>

van der Linden, K., Simons, C., Viechtbauer, W., Ottenheijm, E., van Amelsvoort, T., & Marcelis, M. (2021). A momentary assessment study on emotional and biological stress in adult males and females with autism spectrum disorder. *Scientific Reports*, *11*, Article 14160. <https://doi.org/https://dx.doi.org/10.1038/s41598-021-93159-y>

van Oosterhout, J., van der Linden, K., Simons, C. J. P., van Amelsvoort, T., & Marcelis, M. (2022). Exploring the autism spectrum: Moderating effects of neuroticism on stress reactivity and on the association between social context and negative affect. *Development & Psychopathology*, *34*(4), 1366-1375. <https://doi.org/10.1017/S0954579420002278>
