# Supplementary figures and images for: A systematic review of ecological momentary assessment in autism research
Source: Autism. 2024 Dec 18;29(6):1374–89. doi: 10.1177/13623613241305722 (PMC12089685; doi:10.1177/13623613241305722)

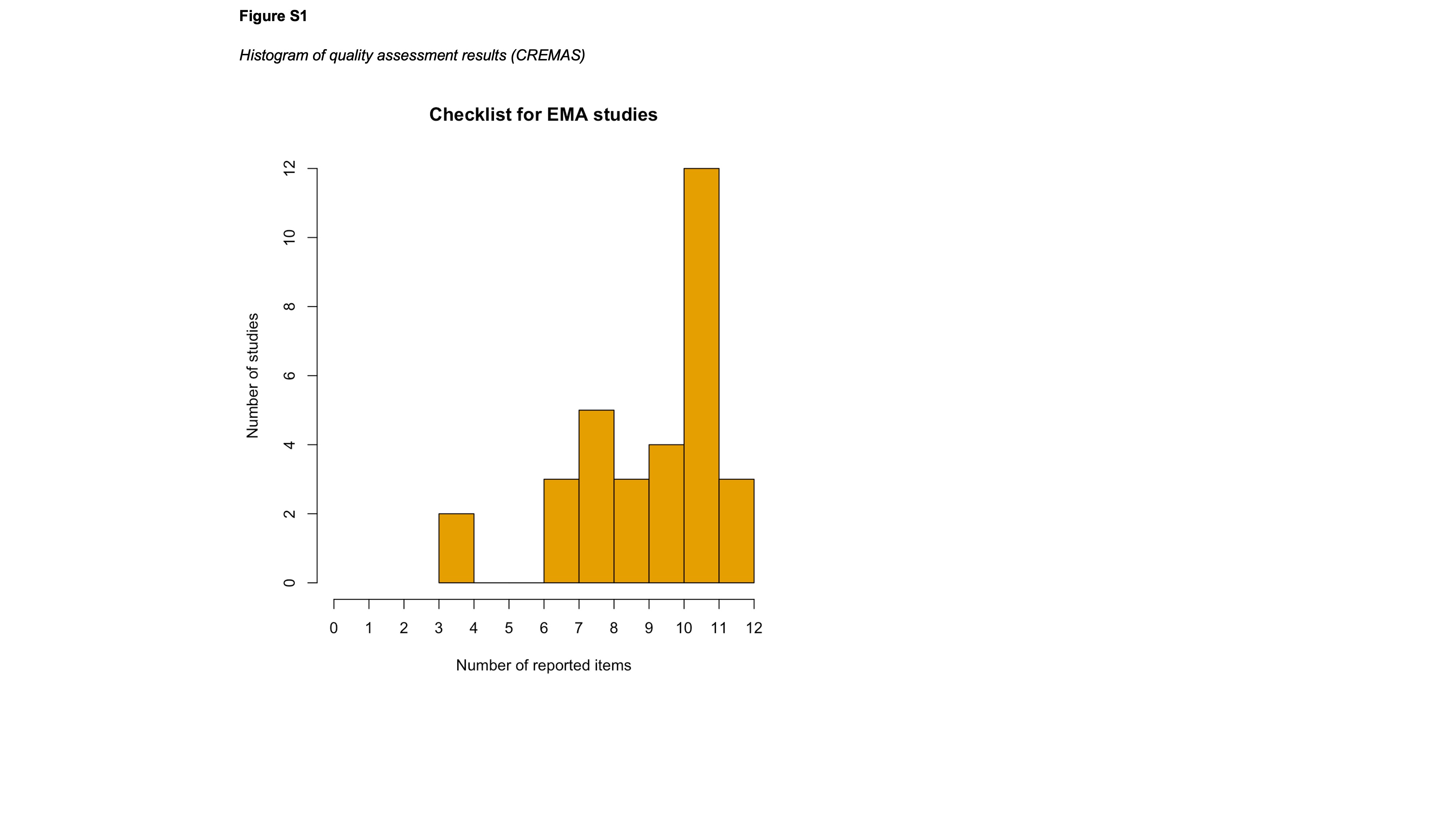

Supplement: sj-jpg-4-aut-10.1177_13623613241305722 – Supplemental material for A systematic review of ecological momentary assessment in autism research [file sj-jpg-4-aut-10.1177_13623613241305722.jpg]

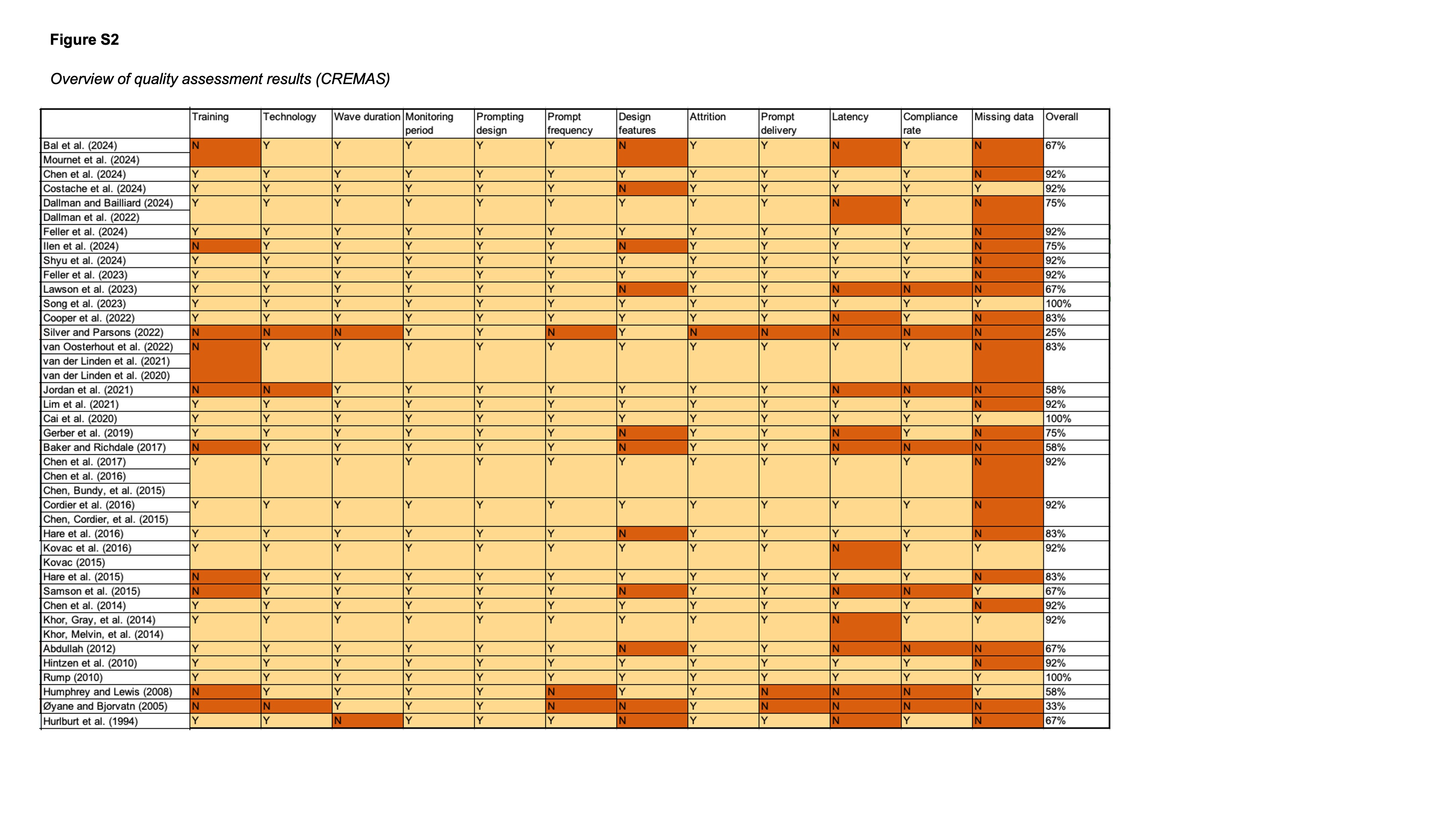

Supplement: sj-jpg-5-aut-10.1177_13623613241305722 – Supplemental material for A systematic review of ecological momentary assessment in autism research [file sj-jpg-5-aut-10.1177_13623613241305722.jpg]
